# An Evaluation of Medical Students' Perceptions, Knowledge, and Attitudes About People With Disability After Attending the Learning Session on Disability Competency: A Cross-Sectional Study

**DOI:** 10.7759/cureus.53878

**Published:** 2024-02-08

**Authors:** Manoj K Saurabh, Amit Ranjan, Tejas K Patel

**Affiliations:** 1 Pharmacology and Therapeutics, All India Institute of Medical Sciences, Gorakhpur, Gorakhpur, IND; 2 Physical Medicine and Rehabilitation, All India Institute of Medical Sciences, Gorakhpur, Gorakhpur, IND

**Keywords:** attitude, student’s perception, orientation and foundation course, disability, competency based undergraduate curriculum

## Abstract

Background and objective

The Medical Council of India [now replaced by the National Medical Commission (NMC)] has implemented a new competency-based curriculum for medical education. Eight competencies in the curriculum are related to the principles of disability-inclusive compassionate care. This study aimed to evaluate the knowledge, perceptions, and attitudes among undergraduate medical students about people with disability after attending learning sessions on disability competency.

Materials and methods

After they attended the learning session during the foundation course, participants were evaluated by using a questionnaire involving 26 questions, of which 17 were based on the Likert scale to assess general perceptions towards the person with a disability, while three questions aimed to assess attitudes, and six closed-ended questions tried to assess knowledge about disability.

Results

In the present study, 79.7% (n=157) of the students thought that people with disabilities faced problems getting involved in society, and 81.2% (n=160) felt that it was harder for them to make friends than others. The majority of the students disagreed with the idea that people with disabilities are a burden on society (n=149, 75.6%) or their families (n=119, 60.4%); 65% (n=128) of the students thought that people with disabilities are more determined than others to reach their goals and achieve more owing to their disability (n=104, 52.85%). A total of 161 (81.7%) students disagreed with the statement that people with disabilities should not be optimistic about their future. A comparison of the pre- and post-test data revealed that students' knowledge regarding disability increased and they gained a more positive attitude towards people with a disability after attending teaching and learning sessions (p<0.0001).

Conclusion

Our findings showed a significant improvement in the undergraduate medical students' understanding and empathy toward individuals with disabilities following sessions on disability competency. Teaching and learning sessions on disability competencies for newly admitted students in medical school can sensitize, orient, increase knowledge, and develop positive attitudes toward people with disabilities. Further studies on the topic are needed involving different phases of clinical teaching.

## Introduction

India is a signatory to the United Nations Convention on the Rights of Persons with Disabilities (UNCRPD), and hence it has amended its disability legislation to incorporate a human rights approach. The Rights of Persons with Disabilities (RPWD) Act, 2016 ensures that the rights of persons with disabilities are included in the curriculum of universities, colleges, and schools. Section 39(2)(f) of the Act specifically addresses the inclusion of disability-related content [1]. It mandates the inclusion of disability-related content in all professional courses, including the medical field. Disability is part of human diversity [2]. Any stream or field that deals with human life needs to comprehend and recognize people who have different abilities. The World Health Organization’s World Report on Disability states that while people with disabilities have the same general healthcare needs as others, they are two times more likely to find healthcare providers' skills and facilities inadequate, three times more likely to be denied healthcare, and four times more likely to be treated badly in the healthcare system [3]. These facts necessitate that an Indian medical graduate should have disability competence, which is the set of skills and attributes essential to providing quality healthcare to patients with disabilities.

It is the social responsibility of medical institutions to be empathetic toward the marginalized sections of society. The National Medical Commission (NMC) has included eight competencies in the foundation course for undergraduate medical education, which is a mandatory part of the new competency-based curriculum implemented in August 2019 [4]. Hence, all medical colleges under NMC have included disability competency in their foundation courses [5]. Before 2019, the school curriculum and undergraduate medical curriculum did not incorporate disability competency. This study aimed to assess the knowledge, perceptions, and attitudes among undergraduate medical students about individuals with disabilities following sessions on disability competency. We believe our findings will contribute to the provision of enhanced and more inclusive healthcare services, ultimately leading to an overall improvement in the healthcare of a marginalized demographic in society.

## Materials and methods

This cross-sectional study was conducted in the Department of Pharmacology in collaboration with the Department of Physical Medicine and Rehabilitation (PMR) at a tertiary care institute in India and involved a sample size of 200 students who were enrolled in their first year of undergraduate medical education. No formal sample size calculation was performed. All students who attended learning sessions on disability competency were invited to participate in the study. Students who did not respond to the questionnaire fully were excluded from the study. The study was approved by the Institute Human Ethics Committee (approval no: IHEC:BHR/13/2020). The consent waiver was granted as the study falls under the "less than minimum" risk type, implying that the probability of harm or discomfort is nil or not expected and learning sessions were conducted as a part of routine teaching.

A four-hour teaching-learning session on disability competencies was conducted, which included an interactive lecture, a video of how medical and paramedical staff treat people with disabilities, narration by people with disabilities, and various positive and negative news stories from the newspapers. The session was conducted as per NMC guidelines. We covered all eight competencies, which included disability as per UNCRPD, comparing and contrasting the medical and social models of disability, and the RPWD Act 2016. This encompassed verbal and nonverbal empathetic communication techniques while communicating with people with disabilities, social inclusion by raising awareness of the human rights of persons with disabilities, and discussion about universal design. After the learning session, participants were distributed case record forms comprising a total of 26 questions. It included four questions on inclusion, four on discrimination, five on gains, and four on prospects. There were three questions to assess attitudes, and six closed-ended questions to assess knowledge about disability. Seventeen questions were based on a Likert scale to assess a general person's perception of a person with a disability. Sixteen validated questions were from previously published literature, and some modifications were made as per the need for the study [5,6].

**Figure 1:**
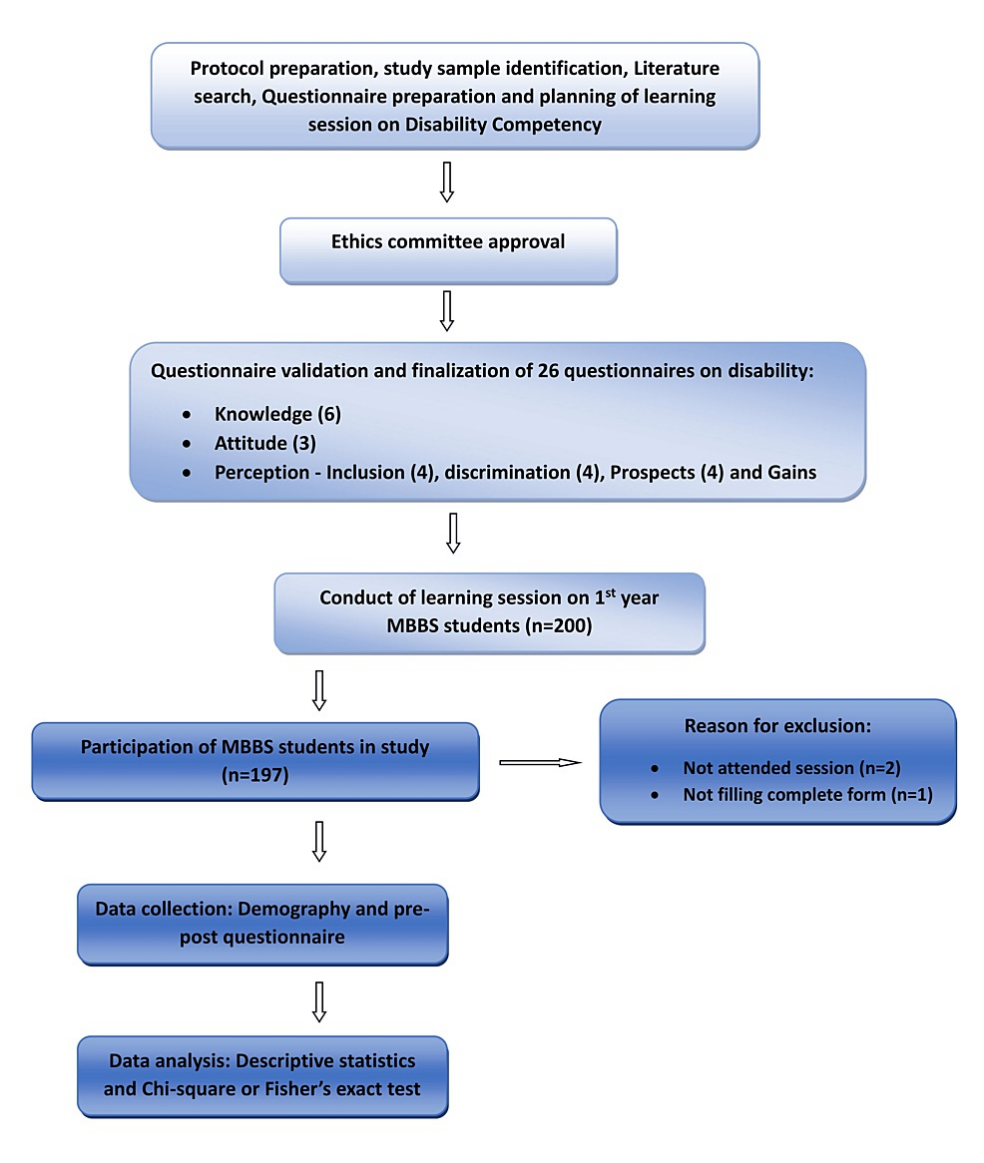
Flow chart depicting the study process

A discussion with faculty members helped to prepare and validate the questionnaire. The final version of the questionnaire was validated using Cronbach's alpha, with a value of 0.86 indicating high internal consistency. The students were also asked to fill in their roll numbers, age, and gender on the case record form. For Likert scale-based questions, students had to select from the following choices of answers: "strongly disagree, disagree, undecided, agree, and strongly agree," with scores ranging from 1 to 5, where 1 corresponded to "strongly disagree" and 5 stood for "strongly agree". The data analysis was performed using descriptive statistics and presented using percentages. The pre- and post-questionnaires were compared using the Chi-square or Fisher’s exact test. A p-value <0.05 was considered statistically significant. The data were analyzed using the GraphPad Prism software demo version 10.0. The detailed flow chart illustrating the study process is presented in Figure 1.

## Results

This study was conducted among newly admitted medical students who attended teaching-learning sessions on disability competency during the foundation course; 197 of the total 200 students participated in the study. Three students were excluded from the study since they failed to respond to all the queries in the case record form; 98 (49.70%) of the students were male and 99 (50.25%) were females. The mean age of students was 17.5 ±0.41 years The sociodemographic profile of students is presented in Table 1.

**Table 1:** Sociodemographic profile CBSE: Central Board of Secondary Education

Characteristics	Number (N=200)	Percentage (%)
Total number of included participants	197	98.5
Male	98	49.7
Female	99	50.25
CBSE Board	73	37.05
Gujarat State Board	124	62.94
English Medium	141	71.57
Gujarati Medium	56	28.42

Of note, 79.7% (n=157) of the students believed that people with disabilities have problems getting involved in society, and 81.2% (n=160) stated that it is harder for them to make friends than others; 75.6% (n=149) of the students expressed dissent towards the notion that individuals with disabilities impose a burden on society, while 60.4% (n=119) disagreed with the idea that such individuals are a burden on their families. Also, 67.5% (n=133) of students thought that people often make fun of disabilities, but only 44.7% (n=88) agreed with the idea that people with a disability are easier to take advantage of (exploit or treat badly) compared with other people, and the remaining were either neutral (20.3%, n=40) or disagreed (35%, n=69). A fair proportion believed that people tend to become impatient with those with disabilities (46.7%) and have no feelings (30.5%). This indicates that a person with a disability may be discriminated against in society. The survey data reflects that 56.3% (n=111) and 41.6% (n=82) of newly admitted students felt that disability could make someone a stronger and wiser person, respectively.

Significantly, 65% of students thought that people with disabilities were more determined than others to reach their goals and achieve more due to their disability (52.85%), as they are more focused on their goals; 45.7% (n=90) of students expressed their disagreement with the notion that students with a level of disability exceeding 80% should be excluded from admission to the medical course. The widespread belief that doctor schedules are often highly demanding and hectic, making it difficult for someone with more than 80% disability to keep up with them, could be the cause of this. However, this belief overlooks the fact that sufficient measures and support systems can be put in place to ensure equal opportunities for all students, regardless of their disabilities. These measures and support systems can help level the playing field and enable students with disabilities to thrive academically. Only 31% (n=61) and 36.5% (n=72) of students agreed that sex should not be discussed with people with disabilities and that people should not expect too much from them, respectively; about 81% (161) of students disagreed with the statement that people with a disability should not be optimistic about their future. Also, 71.6% (n=141) of students disagreed with the notion that people with disabilities have less to look forward to than normal individuals. The above-mentioned data are presented in Table 2. Gender did not affect perceptions (inclusion, discrimination, gains, and prospects) of undergraduate medical students towards persons with disability (p>0.05).

**Table 2:** Perceptions (inclusion, discrimination, gains, and prospects) of undergraduate medical students toward people with disability

Sl. no.	Questionnaire statements	Agree		Neutral		Disagree	
		Number	Percentage	Number	Percentage	Number	Percentage
1	People with disabilities find it harder than others to make new friends	160	81.2	24	12.2	13	6.6
2	People with disabilities have problems getting involved in society	157	79.7	24	12.2	16	8.1
3	People with disabilities are a burden on society	14	7.1	34	17.3	149	75.6
4	People with disability are a burden on their families	35	17.8	43	21.8	119	60.4
5	People often make fun of disabilities	133	67.5	27	13.7	37	18.8
6	People with disabilities are easier to take advantage of (exploit or treat badly) compared with other people	88	44.7	40	20.3	69	35.0
7	People tend to become impatient with those with disabilities	92	46.7	52	26.4	53	26.9
8	People tend to treat those with disabilities as if they have no feelings	60	30.5	46	23.4	91	46.2
9	Having a disability can make someone a stronger person	111	56.3	47	23.9	39	19.8
10	Having a disability can make someone a wiser person	82	41.6	63	32.0	52	26.4
11	Some people achieve more because of their disability (e.g., they are more successful)	104	52.8	53	26.9	40	20.3
12	Students with more than >80% disability should be permitted to join medical college under the disability quota	90	45.7	50	25.4	57	28.9
13	People with a disability are more determined than others to reach their goals	128	65.0	34	17.3	35	17.8
14	Sex should not be discussed with people with disabilities	61	31.0	84	42.6	48	24.4
15	People should not expect too much from those with disabilities	72	36.5	33	16.8	92	46.7
16	People with a disability should not be optimistic about their future	27	13.7	9	4.6	161	81.7
17	People with disability have less to look forward to than others	33	16.8	23	11.7	141	71.6

Table 3 shows a comparison between correct pre- and post-test responses to questions on knowledge and attitude in the questionnaires on disability. Pre-test data demonstrated that first-year students had generally a poor idea about the number of disability competencies included in the foundation course and a poor understanding of universal design. Only 13.2% (n=26) of them had an understanding of the universal design and only 3% (n=6) could differentiate between social and mental disabilities. Only 5.6% (n=11) of students were aware of disability etiquette, but half of the students agreed that disability represents diversity. Eighty-eight students (44.7%) favored the charity approach, while only 18.2% advocated the human rights approach toward people with disabilities, and others were unable to say anything before attending the session. The post-test data reflected a significant improvement in knowledge and a change in attitude after attending disability sessions. Fisher's exact test or Chi-square test was applied to calculate the p-value. There was no significant difference between male and female undergraduate students regarding correct pre- and post-test responses in the knowledge and attitude questionnaire on disability (p>0.05).

**Table 3:** Comparison between pre- and post-test responses in the knowledge and attitude questionnaire on disability *P-value calculated by Fisher’s exact test or Chi-square test. ^#^Choice of answers to question 1 in the attitude domain. ^$^Choice of answers to question 2 in the attitude domain

Sl. no.	Questionnaire items	Pre-test		Post-test		P-value
		Number	Percentage	Number	Percentage	
1	Number of disability conditions recognized as per the Rights for People with Disability (RPWD) Act 2016	0	0	191	96.9	<0.0001*
2	Understanding of universal design	26	13.2	193	98.0	<0.0001*
3	Ability to differentiate between social and medical disability	6	3.0	188	95.4	<0.0001
4	Number of disability competencies included in the foundation course	0	0	193	98.0	<0.0001*
5	Awareness of disability etiquette	11	5.6	193	193	<0.0001
6	Agreement on “Disability is Diversity”	98	50	174	88.3	<0.0001
7	Survival of the fittest^#^	53	26.9	93	47.2	<0.0001
8	Adopt or perish^#^	67	34.1	72	36.5	0.6732
9	Charity^$^	88	44.7	09	4.6	<0.0001
10	Human rights^$^	36	18.27	175	88.83	<0.0001

## Discussion

Although education about disability was not given much attention earlier, many countries incorporated it into their curriculums in the 21st century. Even in developed countries like the United States, scarce resources are allocated to disability education in medical education programs. Kirschner et al. shed light on it in a study 10 years ago and suggested incorporating six core competencies in professional healthcare education [7]. UNCRPD is aimed at "promoting, protecting, and ensuring the full and equal enjoyment of all human rights and fundamental freedoms by all persons with disabilities and to promote respect for their inherent dignity [8]. Section 47(1)(b) of the Rights of Persons with Disabilities Act 2016 recommends "induct disability as a component for all education courses for schools, colleges, and universities for teachers, doctors, nurses, paramedical personnel, social welfare officers, rural development officers, Accredited Social Health Activist (ASHA) workers, Anganwadi workers, engineers, architects, other professionals, and community workers" [9].

NMC has implemented several measures to address the issue of including components of humanities and disability in the curriculum; however, the 1997 regulations (modified in 2017) were found to be inadequate. The new guidelines in 2018 also did not contain the word "humanities." [10]. Hence, the medical education regulator of the country updated its undergraduate curriculum to a competency-based model to conform to global standards. The creation of an "Indian Medical Graduate" (IMG) with the "requisite knowledge, skills, attitudes, values, and responsiveness so that she or he may function appropriately and effectively as a physician of first contact with the community while being globally relevant" [11] is the explicit goal of the new curriculum, known as the competency-based undergraduate curriculum. It has added eight competencies related to disability to the curriculum of the foundation course [12]. It is based on the idea of AETCOM (Attitude, Ethics, and Communication), and students can also opt to learn it during electives, i.e., after the second professional exam. Additionally, the competency-based undergraduate curriculum aims to ensure that students are well-equipped to support and address the needs of individuals with disabilities in their future careers.

Our search for relevant literature on Google Scholar, Scopus, and PubMed did not reveal any similar types of studies, and hence a comparison is not possible, although many articles have been published on the foundation course. The mean age of the students attending the foundation course in our study was 17.5 ±0.41 years, which is less than that reported in a study from south India, which was 18.14 ±0.79 years [13], and students were sensitized to the skills and attributes essential for providing healthcare to patients with disabilities by visiting a special school. The majority of students felt that patients with disabilities faced difficulty in making friends and being accepted in society, but they were not a burden to society or their families. A study conducted among undergraduate students reported that those who have taken lectures are likely to feel like they can be friends with people with disabilities, work together, or spend time together [14,15]. A person with a disability may be discriminated against in society, and some people make fun of a person with a disability. We have to take appropriate actions to minimize such incidents.

There was a significant difference (p<0001) in scores between the knowledge tests given before and after the event. The knowledge tests asked about the number of disability conditions recognized by the RPWD Act 2016, understanding of Universal Design, the ability to distinguish between social and medical disability, disability etiquette, agreement on "Disability is Diversity," and attitude toward treating a person with a disability with a human rights approach. Singh et al. made an excellent effort to include disability competencies in the current medical curriculum with a human rights approach, as opposed to a charity approach or a medical approach [16]. These aspects were addressed in our study as well.

Limitations

This study has a few limitations. It involved newly admitted undergraduate medical students, with teaching sessions limited to a duration of four hours. An inherent drawback of this design was the absence of a control group. Further limitations pertain to the fact that a total of 17 questions were presented at a particular instance as a component of a cross-sectional examination, excluding inquiries about knowledge and attitude domains. Due to the study's focus on evaluating views and attitudes immediately following the intervention, and the collection of participant roll numbers, there is a potential for response bias.

Scope for future studies

We recommend conducting similar studies in different phases of undergraduate medical teaching, focusing more on psychomotor skills and affective domains during clinical teaching.

## Conclusions

A majority of our participants believed that people with disabilities face challenges in getting involved in society and making friends. However, most of the students disagreed with the notion that people with disabilities are a burden on society and their families. A significant proportion of students also believed that people with disabilities are determined to reach their goals and achieve more. Additionally, the study found that teaching and learning sessions on disability competencies improved the students' understanding and empathy towards individuals with disabilities. Teaching and learning sessions on disability competencies for newly admitted students in medical school can help sensitize, orient, increase knowledge, and foster positive attitudes toward patients with disabilities. Further research on the topic is needed in different phases of clinical teaching.

## References

[1] (2024). Department of Empowerment of Persons with Disabilities. The Rights of Persons with Disabilities Act. 2016. https://www.indiacode.nic.in/bitstream/123456789/15939/1/the_rights_of_persons_with_disabilities_act%2C_2016.pdf.

[2] Sukhramani N, Verma S (2013). Disability as diversity: the Indian perception. Diversity and Respect - Problems of Perception in the Global Agenda for Social Work.

[3] (2024). World Health Organization, The World Bank. World Report on Disability. https://www.who.int/publications/i/item/9789241564182.

[4] Bhuyan A (2024). MCI finally updates MBBS curriculum to include disability rights and dignity. https://thewire.in/health/mci-mbbs-curriculum-disability-rights.

[5] Gohain MP (2024). MBBS students to learn disability rights. https://timesofindia.indiatimes.com/city/delhi/mbbs-students-to-learn-disability-rights/articleshow/70578920.cms.

[6] Zheng Q, Tian Q, Hao C (2016). Comparison of attitudes toward disability and people with disability among caregivers, the public, and people with disability: findings from a cross-sectional survey. BMC Public Health.

[7] Power MJ, Green AM (2010). The Attitudes to Disability Scale (ADS): development and psychometric properties. J Intellect Disabil Res.

[8] Kirschner KL, Curry RH (2009). Educating health care professionals to care for patients with disabilities. JAMA.

[9] (2024). Convention on the Rights of Persons With Disabilities - Articles. https://www.un.org/development/desa/disabilities/convention-on-the-rights-of-persons-with-disabilities/convention-on-the-rights-of-persons-with-disabilities-2.html.

[10] Prabhu G (2019). The disappearing act: humanities in the medical curriculum in India. Indian J Med Ethics.

[11] (2024). Competency-based undergraduate curriculum for the Indian medical graduate. https://www.nmc.org.in/wp-content/uploads/2020/01/UG-Curriculum-Vol-I.pdf.

[12] (2024). Foundation course for the undergraduate medical education program. https://www.nmc.org.in/wp-content/uploads/2020/08/FOUNDATION-COURSE-MBBS-17.07.2019.pdf.

[13] Velusami D, R Dongre A, N Kagne R (2020). Evaluation of one-month foundation course for the first year undergraduate students at a medical college in Puducherry, India. J Adv Med Educ Prof.

[14] Shin GI, Woo YS, Park HY, Kim JR (2017). A study about factors influencing on awareness toward the people with disabilities by undergraduate students. J Relabel Res.

[15] Park YK, Kim JH (2017). Analysis of prejudices and attitudes of students in the department of physical therapy against people with disabilities. J Phys Ther Sci.

[16] Singh S, Cotts KG, Maroof KA, Dhaliwal U, Singh N, Xie T (2020). Disability-inclusive compassionate care: disability competencies for an Indian medical graduate. J Family Med Prim Care.

